# Impact of host demography and evolutionary history on endosymbiont molecular evolution: A test in carpenter ants (genus *Camponotus*) and their *Blochmannia* endosymbionts

**DOI:** 10.1002/ece3.9026

**Published:** 2022-07-03

**Authors:** Joseph D. Manthey, Jennifer C. Girón, Jack P. Hruska

**Affiliations:** ^1^ Department of Biological Sciences Texas Tech University Lubbock Texas USA; ^2^ Department of Entomology Purdue University West Lafayette Indiana USA; ^3^ Natural Science Research Laboratory Museum of Texas Tech University Lubbock Texas USA

**Keywords:** Codiversification, genome evolution, genomics, mutualism

## Abstract

Obligate endosymbioses are tight associations between symbionts and the hosts they live inside. Hosts and their associated obligate endosymbionts generally exhibit codiversification, which has been documented in taxonomically diverse insect lineages. Host demography (e.g., effective population sizes) may impact the demography of endosymbionts, which may lead to an association between host demography and the patterns and processes of endosymbiont molecular evolution. Here, we used whole‐genome sequencing data for carpenter ants (Genus *Camponotus;* subgenera *Camponotus* and *Tanaemyrmex*) and their *Blochmannia* endosymbionts as our study system to address whether *Camponotus* demography shapes *Blochmannia* molecular evolution. Using whole‐genome phylogenomics, we confirmed previous work identifying codiversification between carpenter ants and their *Blochmannia* endosymbionts. We found that *Blochmannia* genes have evolved at a pace ~30× faster than that of their hosts' molecular evolution and that these rates are positively associated with host rates of molecular evolution. Using multiple tests for selection in *Blochmannia* genes, we found signatures of positive selection and shifts in selection strength across the phylogeny. Host demography was associated with *Blochmannia* shifts toward increased selection strengths, but not associated with *Blochmannia* selection relaxation, positive selection, genetic drift rates, or genome size evolution. Mixed support for relationships between host effective population sizes and *Blochmannia* molecular evolution suggests weak or uncoupled relationships between host demography and *Blochmannia* population genomic processes. Finally, we found that *Blochmannia* genome size evolution was associated with genome‐wide estimates of genetic drift and number of genes with relaxed selection pressures.

## Graphical abstract

We used whole‐genome sequencing data for carpenter ants (Genus *Camponotus*; subgenera *Camponotus* and *Tanaemyrmex*) and their *Blochmannia* endosymbionts as our study system to address whether *Camponotus* demography shapes *Blochmannia* molecular evolution. We found that host demography was associated with *Blochmannia* shifts toward increased selection strengths, but not associated with *Blochmannia* selection relaxation, positive selection, genetic drift rates, or genome size evolution. Additionally, we found that *Blochmannia* genome size evolution was associated with genome‐wide estimates of genetic drift and number of genes with relaxed selection pressures.

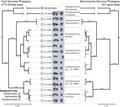

## 1 INTRODUCTION

Symbiotic associations between eukaryotic and prokaryotic organisms are ubiquitous in nature and highly variable (Dimijian, 2000; Moya et al., 2008). Through symbiotic associations with their single‐celled partners, eukaryotes may increase their repertoire of metabolic functions, broadening the range of resources or environments they can exploit (Gil et al., 2004; Moya et al., 2008). Endosymbioses, where one of the symbiotic organisms lives inside the other, are common in insects, may be intra‐ or extracellular, and the mutualisms or commensalisms range in dependence from facultative to obligate (Kikuchi, 2009). Obligate endosymbioses in insects are particularly common and are often implicated in host nutrition and resistance to pathogens (Anbutsu et al., 2017; Brownlie & Johnson, 2009; Moreau, 2020; Perlmutter & Bordenstein, 2020). When obligate, endosymbionts are vertically transmitted from host mothers to their offspring (Bright & Bulgheresi, 2010; Clark et al., 2000); this vertical transmission leads to codiversification between the host and its endosymbiont, and has been demonstrated across insects in the orders Coleoptera, Hemiptera, Hymenoptera, and Lepidoptera, among others (Chen et al., 1999; Clark et al., 2001; Clark et al., 2000; Gil et al., 2004; Gueguen et al., 2010; Heddi et al., 1999; Lo et al., 2003; Moran et al., 2008; Moreau, 2020; Russell et al., 2012; Sauer et al., 2000).

Obligate endosymbionts not only share linked evolutionary histories with their hosts, but endosymbiont demography may also be impacted by host demography (Wernegreen, 2002). Because host and endosymbiont effective population sizes are (at least partially) intrinsically linked, host demographic history may influence the potential strength of selection and rate of genetic drift in endosymbiont genomes. Effective population size is linked with the potential strength of selection and in general, we may expect relaxed selection strengths in relatively smaller population sizes. Additionally, selection in endosymbiont genomes will be partially affected by host‐level selection because endosymbiont fitness is partially linked with host fitness (Wernegreen, 2002). In addition to selection, rates of genetic drift are strongly linked with effective population sizes. Asexual organisms—including bacterial endosymbionts—are also subject to accumulation and fixation of deleterious mutations due to their lack of recombination during reproduction (Moran, 1996; Muller, 1964; Pettersson & Berg, 2007). In a simulation study, Rispe and Moran (2000) showed that endosymbiont mutation fixation rate was higher in relatively smaller host populations. However, endosymbiont population sizes could also be decoupled from host population sizes. For example, endosymbiont transmission population bottlenecks (Mira & Moran, 2002) that vary in different host populations or species would lead to variance in the relationship between host and endosymbiont population sizes. Additionally, endosymbionts may be subject to within‐host selection (Perreau et al., 2021) that could differentially change endosymbiont population sizes in different host populations. Despite the potential for host demography to shape endosymbiont molecular evolution, this topic has been largely unexplored in wild host and obligate endosymbiont systems.

The symbiotic relationship between carpenter ants (genus *Camponotus* Mayr, 1861) and their *Blochmannia* bacterial endosymbionts is an ideal system for investigating the influence of host demographic history on its associated endosymbionts. *Camponotus* is the second largest ant genus with over 2000 species grouped in 45 subgenera (AntWeb, 2022; Ward et al., 2016); these ants are common in woodlands across most of the world (Mackay, 2019; Wilson, 1976). *Camponotus* belongs to the formicine tribe Camponotini that is composed of eight extant genera (Ward et al., 2016). Camponotines have maintained a relationship with *Blochmannia* for about 40 million years (Wernegreen et al., 2009); *Blochmannia* is a vertically transmitted, obligate intracellular bacterial symbiont (Ward et al., 2016) that was first recognized during the late 1800s (Blochmann, 1892). *Blochmannia* are found in specialized cells (bacteriocytes) associated with host midgut tissue and found in the ovaries and oocytes of reproductive females (Ramalho et al., 2018; Wernegreen et al., 2009; Wolschin et al., 2004). *Blochmannia* provide amino acids to their hosts (Feldhaar et al., 2007), and, consistent with a long‐term endosymbiosis, there is evidence that *Camponotus* and *Blochmannia* have histories of co‐speciation because the evolutionary history of the symbionts reflects that of the ants (Degnan et al., 2004; Sauer et al., 2000; Wernegreen et al., 2009).

Several aspects of the *Camponotus*–*Blochmannia* relationship have been studied in detail, including location of the symbionts in the host body (Kupper et al., 2016), transmission method (Ramalho et al., 2018), relative abundance and transcriptional variation across host developmental stages (Ramalho et al., 2017; Stoll et al., 2009), the effects of host development and reproduction on symbiont replication (Wolschin et al., 2004), and the endosymbiont's beneficial role in host nutrition (Feldhaar et al., 2007). One aspect of the *Camponotus*‐*Blochmannia* relationship that remains poorly known is how host evolutionary and demographic histories affect endosymbiont molecular evolution. A few studies to date have examined the evolution of entire *Blochmannia* genomes; in *Blochmannia vafer*, there is some evidence of ongoing purifying selection (Williams & Wernegreen, 2012), and in comparative analyses across three *Blochmannia* genomes, gene loss patterns differ across lineages, suggestive of differential selective pressures in different host lineages (Williams & Wernegreen, 2015).

**FIGURE 1:**
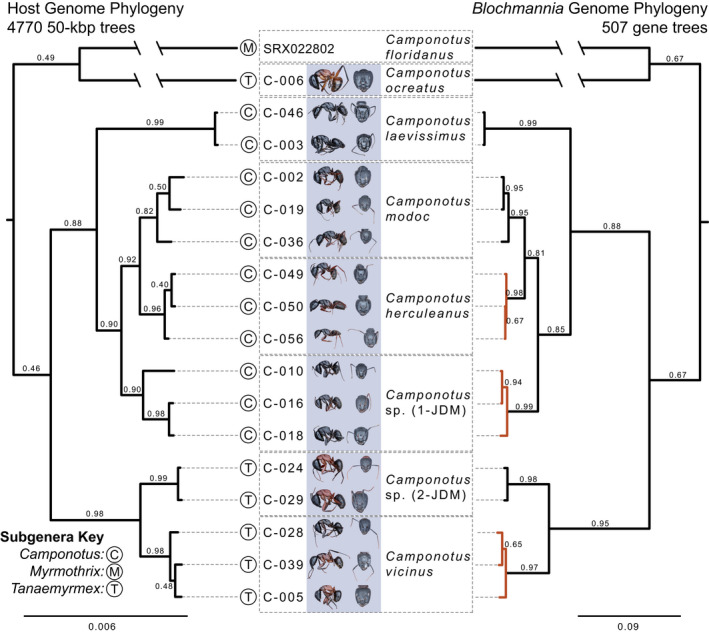
Phylogenetic congruence of host ants and their *Blochmannia* endosymbionts. Branch labels indicate proportion of trees supporting this phylogenetic hypothesis. The ASTRAL species tree topologies were identical to these phylogenies and exhibited 100% quartet support for every relationship. Host trees were rooted with the *Cataglyphis nigra* sample. The *Blochmannia* tree was midpoint rooted. Orange branches in the *Blochmannia* tree indicate branches that vary between the host and endosymbiont phylogenies. Ant photos by JCG and JDM

Here, we aimed to study how *Camponotus* demography and evolutionary history impacts molecular evolution in *Blochmannia* endosymbionts using data from seven *Camponotus* species (Figure 1; Table 1). Our study taxa include seven species from two subgenera in the genus *Camponotus*: *Tanaemyrmex* Ashmead, 1905 (*N* = 3 species) and *Camponotus* (*N* = 4 species). All the species included here are large ants (major workers >1 cm) associated with woodlands in western North America (Table 1; Table 2). Western North American species of the subgenus *Tanaemyrmex* tend to nest in the soil including soil under rocks or logs while the subgenus *Camponotus* tends to nest in decaying logs and stumps (Mackay, 2019). Carpenter ants are omnivorous; their feeding is comprised of opportunistic predation and foraging of animal and plant‐derived resources (Mackay, 2019) and they are thought to have nitrogen‐poor diets (Moreau, 2020). Because *Blochmannia* provide essential amino acids and may play a role in nitrogen recycling within their hosts (Feldhaar et al., 2007), we may expect species‐ or environment‐specific selective pressures—both increased or relaxed selection—on endosymbiont function in our focal species, and that these selective pressures may be influenced by host demography. To address how *Camponotus* demography and evolutionary history shape *Blochmannia* evolution, we assembled a de novo *Camponotus* genome and 17 de novo *Blochmannia* genomes, as well as resequenced genomes for 17 *Camponotus* individuals. We aimed to address the following questions: (1) Do hosts and endosymbionts exhibit strict phylogenomic codiversification histories? (2) How fast do *Blochmannia* genes evolve and is there rate variation across the genome? (3) Does the rate of host evolution impact the rate of *Blochmannia* evolution? (4) Does host demography shape the strengths of natural selection and genetic drift in endosymbiont genomes?

**TABLE 1:** Carpenter ant host and *Blochmannia* endosymbiont characteristics

Species	Host characteristics		*Blochmannia* genome characteristics				
	N_e_*	H_O_*	Genome‐wide K_a_ / K_s_	Genes increased selection	Genes relaxed selection	Gene count*	Genome size*
*C. herculeanus*	30.58	0.2021	0.238	28	13	599	790,985
*C. laevissimus*	10.09	0.0638	0.277	9	29	578	783,856
*C. modoc*	22.42	0.1637	0.218	15	18	600	790,461
*C*. sp. (1‐JDM)	14.11	0.0879	0.195	18	10	598	789,892
*C*. sp. (2‐JDM)	13.88	0.1655	0.219	21	16	597	780,578
*C. vicinus*	40.37	0.2393	0.207	42	29	596	776,986

*Note*: An asterisk (*) indicates calculations that were measured per individual and reported here as the mean per species. N_e_ = harmonic mean effective population size; H_O_ = observed heterozygosity (×100).

**TABLE 2:** Sampling information for the *Camponotus* individuals used in this study

Catalog #	ID	Subgenus	Species	Lat.	Long.	Elev.	Region	Nest found or group foraging?
TTU‐Z_268552	C‐002	*Camponotus*	*modoc*	32.667	−109.873	2761	Pinaleño	large log
TTU‐Z_268553	C‐003	*Camponotus*	*laevissimus*	32.634	−109.816	2226	Pinaleño	large log
TTU‐Z_268554	C‐005	*Tanaemyrmex*	*vicinus*	31.43	−110.29	2012	Huachuca	found foraging at night
TTU‐Z_268555	C‐006	*Tanaemyrmex*	*ocreatus*	31.726	−110.875	1513	Santa Rita	found foraging at night
TTU‐Z_268556	C‐010	*Camponotus*	sp. (1‐JDM)	32.419	−110.735	2486	Santa Catalina	dried out old small stump
TTU‐Z_268557	C‐016	*Camponotus*	sp. (1‐JDM)	33.812	−109.159	2589	White Mountains	large pine stump
TTU‐Z_268558	C‐018	*Camponotus*	sp. (1‐JDM)	34.327	−110.832	2303	White Mountains	large burned pine log
TTU‐Z_268559	C‐019	*Camponotus*	*modoc*	34.369	−110.996	2390	Central Mogollon	large log
TTU‐Z_268560	C‐024	*Tanaemyrmex*	sp. (2‐JDM)	34.909	−111.529	2252	Central Mogollon	found foraging at night
TTU‐Z_268561	C‐028	*Tanaemyrmex*	*vicinus*	35.928	−111.914	2279	South Rim	nest in soil
TTU‐Z_268562	C‐029	*Tanaemyrmex*	sp. (2‐JDM)	35.928	−111.913	2278	South Rim	found foraging at night
TTU‐Z_268563	C‐036	*Camponotus*	*modoc*	36.529	−112.177	2672	North Rim	large pine stump
TTU‐Z_268564	C‐039	*Tanaemyrmex*	*vicinus*	36.381	−112.35	2325	North Rim	found foraging at night
TTU‐Z_268565	C‐046	*Camponotus*	*laevissimus*	37.923	−109.488	2395	Manti La Sal	dried pinon log
TTU‐Z_268566	C‐049	*Camponotus*	*herculeanus*	38.508	−109.29	2464	Manti La Sal	small aspen log
TTU‐Z_268567	C‐050	*Camponotus*	*herculeanus*	38.526	−109.281	2815	Manti La Sal	adjacent large logs
TTU‐Z_268568	C‐056	*Camponotus*	*herculeanus*	38.401	−108.325	2846	Uncompahgre	base of small stump

*Note*: ID and Catalog #s = collection IDs that may be associated with specimens deposited at the Texas Tech Natural Science Research Laboratory.

## 2 METHODS

### 2.1 Field work

We collected *Camponotus* ant specimens from 17 colonies for this study in summer 2018 from Arizona, Colorado, and Utah, USA (Table 2). We actively searched for *Camponotus* colonies by spotting woody debris during the day and tracking *Camponotus* activity during the night. Specimens were placed in cryotubes and frozen with dry ice in the field. Specimens were identified using keys (Mackay, 2019) or comparison with available sequences on NCBI's GenBank. All specimen collection numbers (Table 2) and throughout the manuscript are associated with voucher specimens housed in the Invertebrate Zoology Collection of the Natural Science Research Laboratory, Museum of Texas Tech University. One individual of each of the sequenced colonies was mounted and photographed using a Macropod Pro 3D system (see ant pictures in Figure 1).

### 2.2 *Camponotus* de novo genome assembly and annotation

As of January 2022, the only available genome of a *Camponotus* species is that of *Camponotus* (*Myrmothrix*) *floridanus* (Buckley, 1866) (Genome Accession: GCA_003227725.1 Cflo_v7.5). We decided to assemble the genome of a member of the subgenus *Camponotus* because our research group is focusing on this subgenus in this and several other projects. In April 2022, a somewhat contiguous genome of *C. pennsylvanicus* (a member of the subgenus *Camponotus*) was reported in a preprint (Faulk, 2022); our genome is assembled into scaffolds ~10× more contiguous than that of this reported *C. pennsylvanicus* genome (Table 3).

**TABLE 3:** *Camponotus* sp. (1‐JDM) genome assembly statistics as output by the bbmap stats.sh script

Statistic	CaSp_TTU_1.0
# scaffolds / contigs	695/1738
Largest scaffold / contig	28.327 Mbp / 2.935 Mbp
Scaffold / contig N50	11/164
Scaffold / contig N90	29/785
Scaffold / contig L50	10.362 Mbp/501.17 kbp
Scaffold / contig L90	3.583 Mbp/73.89 kbp
GC (%)	34.46

#### 2.2.1 Genome sample and sequencing

For the reference genome, we chose a North American species in the subgenus *Camponotus* that is currently undescribed (based on phylogenomic analyses; unpublished results). We refrain from naming this reference genome species until we complete a thorough genomic and morphological analysis in the future that includes all *Camponotus* species in the *Camponotus* subgenus described from the USA and Canada. Hereafter in the manuscript, we refer to this species with the code name *Camponotus* sp. (1‐JDM). We extracted DNA from four individuals from a single colony of *Camponotus* sp. (1‐JDM) for use in two sequencing methods for genome assembly: (1) long reads with Pacific Biosciences (PacBio) sequencing, and (2) a Hi‐C library sequenced with Illumina technology.

For the PacBio sequencing, we used services of RTL Genomics (Lubbock, TX, USA). They performed a high molecular weight DNA extraction using Qiagen's (Hilden, Germany) MagAttract HMW DNA Kit. A single major worker, with legs and gaster removed, was used for the extraction. The extracted DNA was then used for PacBio SMRTbell library preparation, size selection using a Blue Pippin (Sage Science), and sequencing on four PacBio Sequel SMRTcells 1 M v3 with Sequencing 3.0 reagents. We used the services of the Texas A&M University Core Facility to prepare a Hi‐C library. They used a single major worker as input for the Arima Genomics Hi‐C kit (San Diego, CA, USA). The Hi‐C library was then sequenced on a partial lane of an Illumina NovaSeq S1 flow cell at the Texas Tech University Center for Biotechnology and Genomics.

#### 2.2.2 Genome assembly and annotation

We assembled the *Camponotus* sp. (1‐JDM) genome in two stages. First, we used Canu v1.9 (Koren et al., 2017) to de novo assemble the PacBio long reads. Second, we used the Hi‐C sequence data to scaffold the initial assembly using the 3D‐DNA pipeline (Dudchenko et al., 2017; Durand et al., 2016). All commands for the assembly, annotation, and all further analyses are documented on GitHub (github.com/jdmanthey/camponotus_genomes1). We quality checked the genome assembly for potential contamination with BlobTools v.1.0.1 (DOI:10.5281/zenodo.845347; Laetsch & Blaxter, 2017). Briefly, BlobTools attempts to identify contamination through taxonomic annotation and coverage parsing of resequencing data to the reference genome. With the use of BlobTools, we identified ~37 kbp of potential contamination attributed to chordates or mollusks that we subsequently removed from our assembly.

We used a multistep process to annotate transposable elements (TEs) and repetitive elements in the *Camponotus* sp. (1‐JDM) genome: (1) identify de novo repeats and over‐represented sequences, (2) manually curate repetitive elements, and (3) mask the genome with these elements to create a TE and repetitive element summary file. First, we used RepeatModeler's (v1.0.11; A. F. Smit & Hubley, 2008) implementations of RepeatScout, RECON, and Tandem Repeats Finder to identify repeats based on homology, structure, and repetitiveness in the de novo assembly (Bao & Eddy, 2002; Benson, 1999; Price et al., 2005). We refined the RepeatModeler output by filtering matches to closely related sequences in the RepBase invertebrate database v24.03 (Jurka et al., 2005) and then creating consensus sequences of novel repetitive elements.

First, we removed any RepeatModeler output sequences ≥98% identical to RepBase sequences. Second, we used BLAST and bedtools (Camacho et al., 2009; Quinlan & Hall, 2010) to extract genomic regions matching repetitive elements as well as 1000 bp flanking sequences. We used these extracted sequences to develop consensus sequences for novel TEs using the following steps: (1) alignment using MAFFT (Katoh & Standley, 2013) implemented in Geneious (BioMatters Ltd), (2) 50% majority consensus sequences in Geneious, and (3) trimming any ambiguous nucleotides on the ends of newly created consensus sequences. For any incomplete consensus sequences where we did not recover TE endpoints, we repeated this prior process up to two times. In addition to identifying de novo repeats and manual curation in the *Camponotus* sp. (1‐JDM) genome, we also repeated this process for the published *Formica selysi* Bondroit, 1918 genome (NCBI: GCA_009859135.1; Brelsford et al., 2020). We added this species to increase the diversity of ant TEs in our database, which has been shown to improve annotations by including TEs that may have been missed in other curated species (Boman et al., 2019). To help with naming some of our de novo TEs, we assessed homology of newly curated sequences to the invertebrate RepBase database using BLAST. Lastly, we used a combination TE library including the RepBase invertebrate database and all newly curated TEs described here for use in RepeatMasker v4.08 (A. Smit et al., 2015). Repeatmasker output included a masked genome and summarized repetitive and TE content in the *Camponotus* sp. (1‐JDM) genome.

To annotate genes in the *Camponotus* sp. (1‐JDM) genome, we used the MAKER v2.31.10 pipeline (Cantarel et al., 2008). First, we used MAKER to predict genes using proteins from other closely related ant species: *Camponotus floridanus* (GCF_003227725.1), *Formica exsecta* Nylander, 1846 (GCF_003651465.1), *Lasius niger* Linnaeus, 1758 (GCA_001045655.1), and *Nylanderia fulva* Mayr, 1862 (GCF_005281655.1) (Bonasio et al., 2010; Dhaygude et al., 2019). We used these initial MAKER predictions to train SNAP and Augustus (Korf, 2004; Stanke & Waack, 2003). Lastly, we used the models trained in SNAP and Augustus in a second iteration of MAKER to predict gene models in the *Camponotus* sp. (1‐JDM) genome. We used BUSCO v3 (Simão et al., 2015) with the Hymenoptera single orthologous gene set (set: odb9) to assess genome assembly completeness.

#### 2.2.3 *Camponotus* mutation rate

We extracted the putative *Camponotus* sp. (1‐JDM) coding sequence (CDS) from the assembly using the MAKER output and bedtools. We downloaded the CDS sequences for *Formica exsecta*, *Lasius niger*, and *Nylanderia fulva* (same versions as proteins) for homology‐based comparisons. We performed a reciprocal BLAST of all species versus *Camponotus* sp. (1‐JDM) using blastn (Camacho et al., 2009) to identify putative homologues across datasets.

To align putative homologues between the four ant species, we used T‐Coffee (Notredame et al., 2000). T‐Coffee translates nucleotide sequences, aligns them using several alignment algorithms, takes the averaged best alignment of all alignments, and back translates the protein alignments to provide a nucleotide alignment for each gene. Before the final back‐translating, we used trimAl (Capella‐Gutiérrez et al., 2009) to remove gaps in the protein alignments.

We tested each gene for selection using gene‐wide and branch‐specific tests for selection in CODEML (Yang, 1997). After correcting significance values for multiple testing using the Benjamini and Hochberg (1995) method, we removed any alignments with evidence for selection. We then extracted and concatenated four‐fold degenerate sites from the alignments (*N* = 806,844) using custom R scripts and the R packages “Biostrings” and “seqinr” (Charif & Lobry, 2007; Pagès et al., 2017). With this alignment of four‐fold degenerate sites, we identified an appropriate model of sequence evolution using jModelTest (Darriba et al., 2012) and used the GTR + I model of sequence evolution in PhyML (Guindon et al., 2010) to estimate a phylogenetic tree.

To put the evolution of the CDS four‐fold degenerate sites in a timed evolutionary context, we downloaded a recent phylogenomic tree of formicine ants (Blaimer et al., 2015) and pruned the tree to the four representative lineages covered by our CDS downloads and novel assembly using the R package “ape” (Paradis et al., 2004) using four species as representatives of those lineages: *Camponotus* (*Myrmentoma*) *hyatti* Emery, 1893, *Nylanderia dodo* (Donisthorpe, 1946), *Formica neogagates* Viereck, 1903, and *Lasius niger*. We used the *Camponotus*‐specific branch length of the four‐fold degenerate sites tree along with divergence time estimates from Blaimer et al. (2015) to obtain an estimate of *Camponotus*‐specific mutation rates.

### 2.3 Resequencing *Camponotus* genomes

#### 2.3.1 Lab work

We resequenced genomes for 17 *Camponotus* individuals from 7 species (Table 2) at moderate sequencing coverage (~10–30×). We used a single individual per colony for sequencing. For each individual, we performed two DNA extractions: (1) gaster and (2) head + mesosoma. We did this because the gaster has a plethora of *Blochmannia* DNA relative to ant DNA (Brown & Wernegreen, 2016; Ramalho et al., 2019). For each extraction, we froze the sample with liquid nitrogen and subsequently pulverized the sample with a sterile mortar and pestle. We then used the pulverized material as input for DNA extraction with QIAGEN (Hilden, Germany) DNeasy blood and tissue kits. We quantified DNA concentrations from the extractions with Invitrogen (Carlsbad, California) Qubit fluorescent quantitation, and pooled the head + mesosoma and gaster extracts at a 0.8:0.2 ratio, respectively, to have good representation of both ant and endosymbiont DNA for sequencing. Genomic DNA extractions were sent to the Texas Tech University Center for Biotechnology and Genomics for standard Illumina shotgun sequencing library creation and subsequent sequencing on a partial lane of an S4 flow cell on the Illumina NovaSeq6000.

#### 2.3.2 Filtering and genotyping

First, we downloaded Illumina sequencing reads from the NCBI SRA of two published datasets to use as outgroups: *Camponotus floridanus* (SRX022802) and *Cataglyphis nigra* (André, 1881) (SRX5650044). With our newly generated data and the downloaded data, we trimmed adapters and quality filtered the raw sequencing data using the bbduk.sh script of the bbmap package (Bushnell, 2014). We then aligned the filtered data to the de novo *Camponotus* sp. (1‐JDM) reference genome with BWA (Li & Durbin, 2009) using the BWA‐MEM command. We used samtools v1.4.1 (Li et al., 2009) to convert the BWA output SAM file to BAM format, and lastly cleaned, sorted, added read groups to, and removed duplicates from each BAM file using the Genome Analysis Toolkit (GATK) v4.1.0.0 (McKenna et al., 2010). We used GATK's functions HaplotypeCaller and GenotypeGVCFs to genotype all individuals for both variant and invariant sites on all scaffolds at least two Mbp in length. We measured the distribution of sequencing coverage using the samtools “depth” command. We used VCFtools v0.1.14 (Danecek et al., 2011) to initially filter all variant and invariant site calls using the following restrictions: (1) genotyped in ≥70% of individuals, (2) minimum site quality of 20, (3) minimum genotype quality of 20, (4) minimum depth of coverage of 5, and (5) maximum mean depth of coverage of 70.

### 2.4 Blochmannia genome assemblies and annotation

With the raw sequencing data, we used the MinYS pipeline (Guyomar et al., 2020) to assemble *Blochmannia* genomes for each sample. MinYS used samples mixed with host and bacterial DNA in a pipeline that allows targeted assembly of bacterial genomes. First, it maps metagenomic reads to a reference genome using BWA. Here, we used a *Blochmannia pennsylvanicus* genome (NC_007292.1; Degnan et al., 2005) as our target. Next, the pipeline assembles these recruited reads using the program Minia (github.com/GATB/minia), followed by gapfilling the contigs using the program MindTheGap (Rizk et al., 2014). Finally, the pipeline simplifies the graphical fragment assembly (GFA) output of MindTheGap. The resulting GFA output was then visualized in Bandage (Wick et al., 2015), regions with multiple paths merged by coverage, and output in FASTA format. This process assembled a circular genome for each of the samples in our study. We also downloaded the sequence and annotation of the *Blochmannia* endosymbiont of *Camponotus floridanus* for use as an outgroup (NC_005061.1; Gil et al., 2003). We used the NCBI prokaryotic genome annotation pipeline (Tatusova et al., 2016) to annotate genes in each of the *Blochmannia* genomes.

### 2.5 *Camponotus* phylogenomics and population genomics

#### 2.5.1 Phylogenomics

We estimated “gene trees” for nonoverlapping 50 kbp sliding windows using RAxML v8.2.12 (Stamatakis, 2014) with the GTRGAMMA model of sequence evolution. From these gene trees (*n* = 4770), we estimated a species tree using two methods: (1) maximum clade credibility tree of all input trees using DendroPy (Sukumaran & Holder, 2010), and (2) the coalescent‐based species tree approach ASTRAL III (Zhang et al., 2018).

#### 2.5.2 Genetic diversity and demography

We estimated genetic diversity for each individual in two ways. First, we estimated observed heterozygosity simply as the proportion of bi‐allelic to total genotyped sites (both invariant and variant) for each individual. Because sequencing depth has the potential to impact estimates of genetic diversity, we also used the program ROHan (Renaud et al., 2019), which uses a Bayesian framework to estimate rates of heterozygosity while accounting for sequencing depth and per‐base quality scores. We found the two estimates to be highly correlated in ingroup samples (*r* = 0.843, *p* << .001), so we consider only raw estimates of heterozygosity hereafter.

To estimate demography for each individual, we used the program MSMC2 v1.1.0 (Schiffels & Durbin, 2014). For use in MSMC, we masked genomic regions not genotyped, as these would otherwise be mistaken for runs of homozygosity. It is relevant to note that MSMC estimates are accurate in panmictic populations, but population structure or changes in connectivity between populations through time may mimic changes in population sizes (Chikhi et al., 2018; Mazet et al., 2016). Because of this, some caution should be used when interpreting raw demographic history results. We largely used the demographic histories to estimate harmonic mean population sizes over the past 200,000 years, which is highly correlated with observed heterozygosity (*r* = 0.850, *p* << .001). When running MSMC, we allowed up to 20 iterations and up to 23 inferred distinct time segments. We performed bootstrap replicates for each individual to see how signal could vary using different genomic regions. For this, we bootstrapped 1 Mbp segments of the genomes, with a total of 10 bootstrap replicates. We decided to use MSMC for each individual rather than aggregating samples per species because of several reasons: (1) uncertainty of population structure among sampling locations, (2) uneven sampling sizes per species, and (3) low certainty with phasing necessary to run the program with multiple individuals, again because of the small sample sizes per species.

MSMC output is presented relative to a species' generation time and mutation rate. We used the mutation rate calculated from the de novo genome assembly as described above. Because there are no good estimates of generation times in *Camponotus* ants, we used a conservative proxy for generation time used in other studies: double the age of sexual maturity (Nadachowska‐Brzyska et al., 2015). In *Camponotus*, previous studies have suggested that the earliest age of queens producing winged reproductives is a minimum of 2 years following colony formation, with the first winged individuals overwintering until the third year (Fowler, 1986; Pricer, 1908). As such, we used 3 years as the age of reproductive maturity and 6 years as the generation time for demographic analyses. This value is generally consistent with generation time estimates of 7–8 years in red harvester ant [*Pogonomyrmex barbatus* (Smith, 1858)] colonies kept in captivity (Ingram et al., 2013).

### 2.6 *Blochmannia* phylogenomics and population genomics

#### 2.6.1 Phylogenomics

We extracted coding (CDS) regions from each *Blochmannia* genome using bedtools. We then used BLAST to match each gene to genes in the outgroup *Blochmannia floridanus* to identify putatively homologous genes from each *Blochmannia* genome. For further analysis, we kept 507 genes present in all samples. We aligned all sequences for each gene using T‐Coffee and trimmed any portions of the alignments not present in all samples using trimAl. Next, we used RAxML v8.2.12 (Stamatakis, 2014) with the GTRGAMMA model to estimate a phylogeny for each of the *Blochmannia* genes. From these gene trees, we estimated a species tree using two methods: (1) maximum clade credibility tree of all input trees using DendroPy (Sukumaran & Holder, 2010), and (2) the coalescent‐based species tree approach ASTRAL III (Zhang et al., 2018).

#### 2.6.2 Tests for selection

We used the HyPhy software package (Pond & Muse, 2005) to test for selection in the *Blochmannia* genes in a phylogenetic framework. We tested for positive selection using aBSREL (M. D. Smith et al., 2015) and we tested for shifts in selection strength across the phylogeny using RELAX (Wertheim et al., 2015). We ran aBSREL in exploratory mode where all branches are tested for positive selection. RELAX requires a set of test branches and reference branches to identify shifts in selection strength. As such, we ran RELAX six times, once for each *Camponotus* species in the study with more than a single individual [i.e., excluding *C*. (*Tanaemyrmex*) *ocreatus* Emery, 1893 and the outgroup].

#### 2.6.3 Genetic drift

For each species' *Blochmannia* genomes, we estimated the rate of nonsynonymous substitutions per site (K_a_) relative to the rate of synonymous substitutions per site (K_s_) using the R package “SeqinR” (Charif & Lobry, 2007). Generally, the K_a_/K_s_ ratio is indicative of the strength of selection in coding genes; in a particular gene, we may expect values much less than one under the effects of purifying selection and values greater than one due to positive selection. However, we expect very low genome‐wide K_a_/K_s_ ratios due to selection maintaining function of most genes in the genome (Kuo et al., 2009); in species with smaller effective population sizes, we expect increased genetic drift and reduced efficacy of selection that may result in accumulation of slightly deleterious mutations and higher estimates of genome‐wide K_a_/K_s_ ratios (Kuo et al., 2009). As such, we used genome‐wide K_a_/K_s_ ratios as a proxy for the strength of genetic drift in each species.

#### 2.6.4 Gene loss

We tested for gene loss in all *Blochmannia* genomes assembled for this study. First, we performed an all‐to‐all protein BLAST (blastp) of all amino acid sequences from coding genes annotated from all samples. From the BLAST analysis results, we tabulated a gene presence/absence matrix for each *Blochmannia* genome (*N* = 607 unique coding genes identified from all samples).

### 2.7 Co‐analyses of *Camponotus* and *Blochmannia* data

To estimate correlations between host and endosymbiont traits while accounting for the evolutionary history of the samples, we analyzed all host and *Blochmannia* traits in the context of phylogenetic independent contrasts (PICs). We estimated PICs for each trait in the R package “ape” (Paradis et al., 2004) which uses the method of Felsenstein (1985). This PIC method assumes Brownian motion of trait evolution and transforms the sampled trait data into statistically independent values (contrasts) that may be used in regressions (Felsenstein, 1985).

#### 2.7.1 Evolutionary rates

We explored evolutionary rates in *Blochmannia* genomes in multiple ways. First, we examined variation in *Blochmannia* gene phylogenies relative to the host species tree. To do this, we calculated the Kuhner and Felsenstein (1994); KF94) distance between the host species tree and *Blochmannia* gene trees, implemented in the R package “ape” (Paradis et al., 2004). Second, we explored rates of evolutionary change in a phylogenetic context by measuring relative branch lengths in the host species tree versus the *Blochmannia* gene trees. We calculated these rates of evolutionary change in three groups: (1) subgenus *Camponotus*, (2) subgenus *Tanaemyrmex* excluding *C. ocreatus* because it is on a long branch by itself, and (3) the combined group of the subgenera *Camponotus* and *Tanaemyrmex*. Third, we measured nucleotide percent identity for all gene alignments, excluding indels, in the same three groups as aforementioned. Fourth, for each sampled individual, we measured the correlation between host and endosymbiont root‐to‐tip distance in the species tree phylogenies.

#### 2.7.2 Demographic influences on drift, selection, and gene loss

For each of the six ingroup species (i.e., excluding *C. ocreatus*), we measured the association between host population sizes and (1) changes in selection strength in *Blochmannia* genes, (2) *Blochmannia* genome‐wide K_a_/K_s_ ratios, (3) number of complete genes found in each *Blochmannia* genome, and (4) *Blochmannia* genome size.

## 3 RESULTS

### 3.1 ***Camponotus*** reference genome characteristics and molecular clock

**FIGURE 2:**
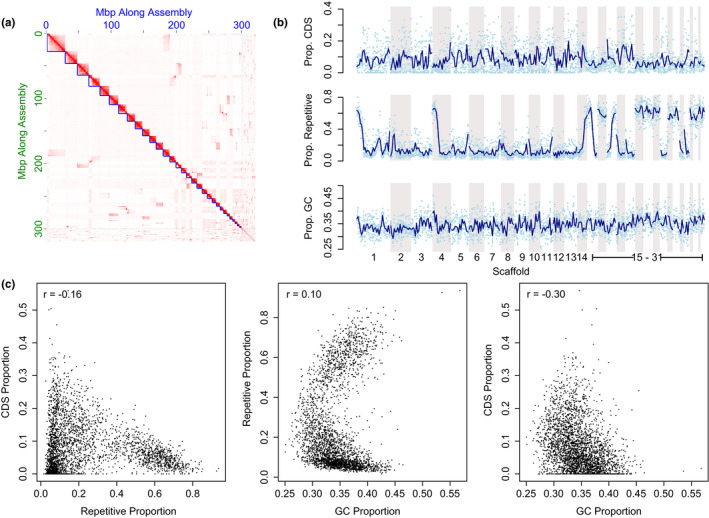
*Camponotus* sp. (1‐JDM) genome assembly characteristics. (a) Hi‐C contact heatmap. Darker colors indicate more read pairs aligning to two regions of the genome. Blue lines indicate scaffold boundaries chosen for the genome assembly. While some regions indicated additional potential contacts—And would further scaffold the genome—We chose to be conservative with combining scaffolds to minimize misassembles. (b) *Camponotus* sp. (1‐JDM) de novo genome assembly content, including coding content (CDS), repetitive and transposable elements, and GC content. Points indicate summary statistics in 100 kbp nonoverlapping sliding windows, while solid lines indicate 10‐window (i.e., 1 Mbp) mean estimates. (c) Relationship between CDS content, repetitive element content, and GC% in 100 kbp sliding windows across the reference genome

Our de novo *Camponotus* sp. (1‐JDM) reference genome was highly contiguous (contig L50 ~ 500 kbp) with a small number of scaffolds composing the majority of the assembly (scaffold L90 ~ 3.58 Mbp, N90 = 29; see Table 3). Overall, we had 31 scaffolds greater than 2 Mbp. Although there are no karyotypes for North American species in the subgenus *Camponotus*, there are estimates for *Camponotus ligniperda* (Latreille, 1802) (haploid *N* = 14), *C. japonicus* Mayr, 1866 (*N* = 13 or 14), and *C. obscuripes* Mayr, 1866 (*N* = 14) from the eastern Palearctic (Hauschteck, 1983; Imai, 1969; Imai & Yosida, 1964). As such, it appears we generated a genome with the contiguity of about two scaffolds per chromosome. While we did have additional signal in the Hi‐C contacts to additionally scaffold the genome (Figure 2a), we chose to be conservative and only link genomic regions where we were confident of the signal (Figure 2a).

The difficulty in fully scaffolding the genome may relate to the repetitive nature of the genome; the genome averaged 24.7% repetitive element content with many large portions of the genome exhibiting greater than 70% repetitive content (Figure 2b). Overall, about 13% of genomic windows contained more than 60% repetitive content. A large proportion of the repetitive content was DNA transposons, both previously described and those manually curated for this study. The repetitive landscape is consistent with other ant species exhibiting “islands” of extreme repetitive content in a background of lower genomic repetitive content (Schrader et al., 2014).

Coding gene content is heterogeneous across the genome and is negatively correlated with both repetitive element content and GC% in 100 kbp sliding windows (Figure 2b,c). BUSCO results suggest our genome is nearly complete and representative of other hymenopterans, containing 98% complete genes and 1.2% fragmented genes of the 4415 hymenopteran near‐universal single‐copy orthologs (Table 4). Using the four‐fold degenerate sites from the genome's CDS regions, we estimated a substitution rate of 1.983877 × 10^−9^ substitutions/site/year.

**TABLE 4:** BUSCO analysis of *Camponotus* sp. (1‐JDM) genome with the hymenoptera_odb9 database, a set of 4415 hymenopteran near‐universal single‐copy orthologs

Complete	4327	98.0%
Complete and single copy	4194	95.0%
Complete and duplicated	133	3.0%
Fragmented	53	1.2%
Missing	35	0.8%

### 3.2 Phylogenomics

We estimated an ant host species tree using 4770 “gene trees” estimated in 50 kbp sliding windows across the genome. Both methods we used to create the species tree—maximum clade credibility and ASTRAL—identified an identical topology (Figure 1). Here, each species was monophyletic. The four species in the subgenus *Camponotus*, formed a monophyletic group; in contrast, most species in the subgenus *Tanaemyrmex* formed a clade, but *C. ocreatus* was recovered as more closely related to *C. floridanus* (subgenus *Myrmothrix*) than to other species in the subgenus *Tanaemyrmex*. Between 40% and 99% of gene trees supported the relationships identified in the species tree, while each node had 100% support in ASTRAL analyses (Figure 1).

**FIGURE 3:**
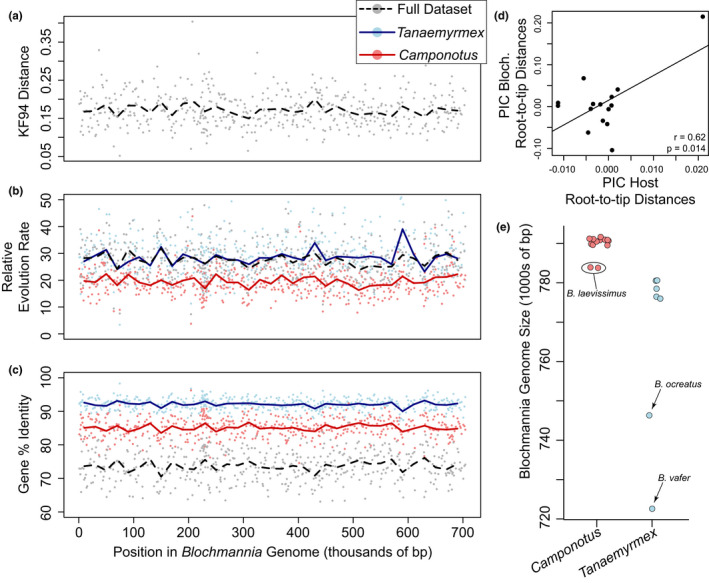
Variation in molecular evolution across the *Blochmannia* genomes. (a) the KF94 distance between the host species tree and *Blochmannia* genes. The KF94 distance measures differences between phylogenetic topologies including branch lengths, where a value of zero is an identical tree. (b) Rates of molecular evolution in *Blochmannia* genes relative to the host species tree. (c) Gene identity percentage in *Blochmannia* genes for alignments with indels removed. For all statistics, lines indicate mean values across windows of 10 genes. (d) Relationship of evolutionary rates between hosts and endosymbionts. PIC = phylogenetic independent contrasts. (e) *Blochmannia* genome assembly sizes for all new assemblies in this study as well as those published on GenBank for these *Camponotus* subgenera. GenBank sequences: *Blochmannia pennsylvanicus* (NC_007292.1), *B. vafer* (NC_014909.2), *B. chromaiodes* (NC_020075.1)

The *Blochmannia* species tree estimated from 507 gene trees identified a strongly supported phylogeny with a nearly identical topology to the host species tree (Figure 1). The only differences were some relationships between individuals within species (colored orange in Figure 1). In contrast to varying proportions of gene trees matching species tree relationships in the ant hosts, a majority of *Blochmannia* gene trees matched relationships of the species tree (67%–99% support for each node). We measured the KF94 distance between each *Blochmannia* gene tree and the host species tree to identify if any regions of the *Blochmannia* genome were relatively discordant from the host phylogenomic signal. In general, the phylogenetic concordance, as measured by the KF94 metric, was consistent across the entire *Blochmannia* genome (Figure 3a).

### 3.3 *Blochmannia* genome sizes, gene composition, rates of molecular evolution

The *Blochmannia* genomes assembled here largely varied per subgenus. In the subgenus *Camponotus*, the genomes varied in size from ~783 to 792 kbp (Figure 3e). This is consistent with *Blochmannia* genomes from this subgenus already on GenBank [*B. pennsylvanicus* (NC_007292.1) = 791 kbp; *B. chromaiodes* (NC_020075.1) = 791 kbp]. In contrast, individuals in the subgenus *Tanaemyrmex* had highly variable *Blochmannia* genome sizes. Five of the genomes ranged in size from 775 kbp to 781 kbp, while *B. ocreatus* was ~746 kbp and a sequence from GenBank for *B. vafer* (NC_014909.2) was ~722 kbp (Figure 3e).

**FIGURE 4:**
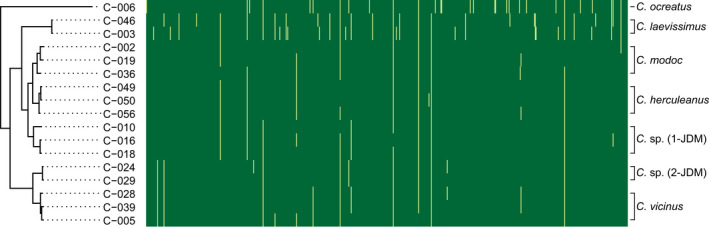
*Blochmannia* gene presence/absence phylogenetic heatmap. Of 607 total genes annotated, 65 varied in the presence/absence among samples. 37 of 65 genes lost in at least one individual exhibit phylogenetic signal. Dark green indicates presence, while light yellow indicates absence

The *Blochmannia* genomes contained between 576 and 601 genes, and in total across all genomes, 607 unique coding genes were annotated. In total, 65 genes exhibited variable presence/absence among the samples sequenced here (Figure 4). Of those 65 genes, 37 exhibited phylogenetic signal of gene loss.

The *Blochmannia* genes evolved at rates ~20–30× faster than the rate of evolution of the ant hosts, with slight variation across the *Blochmannia* genome (Figure 3b). Additionally, it appeared that *Blochmannia* genes in ant hosts of the subgenus *Camponotus* had a slightly slower rate of evolution than those of the ant host subgenus *Tanaemyrmex* (Figure 3b). If we use the *Camponotus* rate of molecular evolution to put *Blochmannia* rates in a timed absolute context, the mean *Blochmannia* gene evolution rate is about 5.474 × 10^−8^ substitutions/site/year (range = 1.454 × 10^−8^ to 1.256 × 10^−7^ substitutions/site/year). Species tree root‐to‐tip distances for host and endosymbionts (trees in Figure 1) showed a significant positive correlation (Figure 3D), suggestive of a genome‐wide molecular evolution association between hosts and their endosymbionts.

*Blochmannia* gene identity within host subgenera was generally consistent across the endosymbiont genome, suggestive of similar evolutionary forces acting across most of the genome at the taxonomic scale of host clades (Figure 3C). We also tried to identify relative rates of evolution and percent sequence identity in intergenic regions. To do this, we performed whole‐genome alignments using progressiveMauve (Darling et al., 2010). However, endosymbiont intergenic sequences were so divergent between host subgenera, and in some cases, between host species, that we were unable to recover any high‐quality alignments in these regions (e.g., large nonoverlapping sections in these regions of the alignments). Needless to say, the rates of evolution in these intergenic regions are likely much higher than the genic rates in Figure 3b.

**FIGURE 5:**
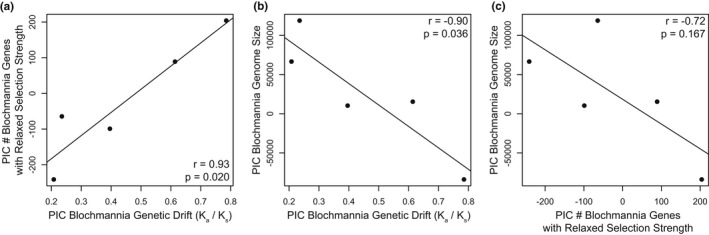
Genome evolution associations in *Blochmannia*. PIC = phylogenetic independent contrast. Shifts in selection strength were obtained using the program RELAX

We found a strong positive association between *Blochmannia* genome‐wide K_a_/K_s_ ratios and number of *Blochmannia* genes with relaxed selection strength (*r* = 0.93, *p* = .020) (Figure 5a). *Blochmannia* genome sizes were associated with both K_a_/K_s_ ratios (*r* = −0.90, *p* = .036) (Figure 5b) and number of *Blochmannia* genes with relaxed selection strength (*r* = −0.72, *p* = .167) (Figure 5c).

### 3.4 Impacts of host demography on endosymbiont evolution

**FIGURE 6:**
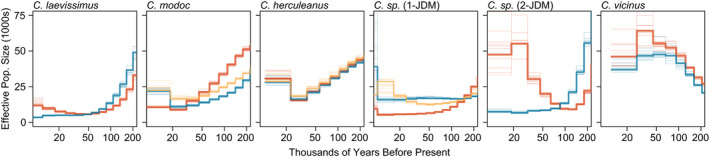
Demographic history estimated with MSMC2. Different individuals are represented with differently colored lines and bootstraps are shown with thin lines

Contemporary estimates of host effective population sizes ranged from ~5000 to 50,000 and, with a couple of exceptions, were largely consistent within species (Figure 6). Variation in demographic histories within species may be indicative of variation in among population gene flow (i.e., a lack of panmixia), and therefore, the overall demographic trends for each individual should be interpreted with this in mind. Overall, however, harmonic mean population size through the last 200,000 years was highly correlated with observed heterozygosity for each individual (*r* = 0.850, *p* << .001), and suggests that the MSMC population size estimates reflect population history, even if not simply population size trends (e.g., variance in estimates due to differential population structure). As such, we looked for correlations between endosymbiont traits and host population sizes using these harmonic mean population size estimates.

**FIGURE 7:**
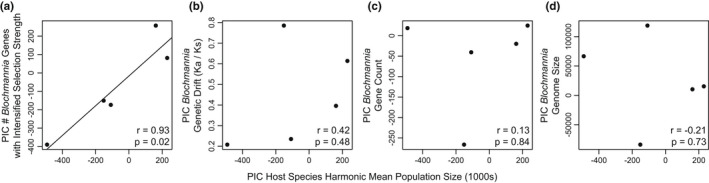
Relationships of host demography and *Blochmannia* molecular evolution. Shifts in selection strength were obtained using the program RELAX. Associations in panels B–D were not significant. PIC = phylogenetic independent contrast

In positive selection tests, 19 *Blochmannia* genes showed evidence for selection. These signatures of positive selection appeared somewhat randomly in the phylogeny (not shown). We also tested for shifts in selection strength (i.e., intensified or relaxed) among the host lineages for all *Blochmannia* genes. We found a positive relationship between host population size estimates and number of endosymbiont genes with shifts toward intensified selection pressures (Figure 7). In contrast, we found no relationship between number of genes with relaxed selection strength and host demography (not shown). As previously mentioned, some gene loss in endosymbiont genomes exhibited phylogenetic signal (Figure 4). Despite this, we found no evidence for a relationship between host population size and gene loss in endosymbiont genomes (Figure 7c). Additionally, we found no significant associations between host population sizes and either *Blochmannia* genome‐wide K_a_/K_s_ ratios (Figure 7b) or *Blochmannia* genome size (Figure 7d).

## 4 DISCUSSION

We sequenced 17 carpenter ant hosts and their *Blochmannia* endosymbionts to address questions about host demography impacts on endosymbiont evolution. We added a whole‐genome resource for one species in the subgenus *Camponotus* and more than doubled the number of publicly available *Blochmannia* full‐genome sequences. With these resources, we investigated questions related to the (1) codiversification of hosts and endosymbionts, (2) molecular evolution of endosymbionts, and (3) effects of host demography on endosymbiont genome patterns of natural selection, drift, size evolution, and gene loss.

### 4.1 Codiversification of carpenter ant hosts and *Blochmannia* endosymbionts

Using whole‐genome sequencing of both carpenter ant hosts and their bacterial endosymbionts, we identified generally strict codiversification (Figure 1). There was some phylogenetic incongruence between host and endosymbiont trees among individuals within species, but all species‐level relationships were completely congruent. These patterns are consistent with expectations of co‐speciation between hosts and vertically transmitted endosymbionts; similar evidence of codiversification between hosts and endosymbionts has been found in bivalves (Distel et al., 1994), weevils (Toju et al., 2013), flies (Chen et al., 1999; Hosokawa et al., 2012), cockroaches (Clark et al., 2001; Lo et al., 2003), aphids (Clark et al., 2000), psyllids (Thao et al., 2000), and previous studies in carpenter ants (Degnan et al., 2004; Sauer et al., 2000). Generally, previous studies investigating codiversification have inferred phylogenies using one or a few molecular markers; in contrast, by sequencing full genomes for both hosts and endosymbionts, we were able to obtain strongly supported species trees as well as estimate variation in lineage sorting across *Blochmannia* genes (Figure 3a) with phylogenetic statistics. Here, we expected one of two patterns: (1) consistent signal across the genome with relatively similar overall rates of evolution across *Blochmannia* genes, or (2) a highly variable landscape of phylogenetic congruence and incongruence caused by variable selective pressures across the genome. Indeed, we found a generally consistent pattern of lineage sorting across the genome as evidenced by a stable estimate of the KF94 statistic across the *Blochmannia* genome.

### 4.2 Rates of molecular evolution in *Blochmannia* endosymbionts

We found that *Blochmannia* genes evolved at a rate ~ 30× faster than the host genome (Figure 3). In addition, intergenic regions were so divergent across lineages that we were not able to align them properly. This endosymbiont–host relative evolution rate is similar to the level reported in *Buchnera* bacterial endosymbionts of aphids (~36×) by Moran et al. (1995). On an absolute scale, we estimated *Blochmannia* genes evolve at ~5.474 × 10^−8^ substitutions/site/year. Degnan et al. (2004) estimated substitution rates at synonymous sites for four *Blochmannia* genes and measured rates (~1.094 × 10^−7^ substitutions/site/year) about twice as fast as our estimates (from all genes from the entire genome). This difference makes sense because we should expect substitution rates that do not lead to amino acid changes to be faster relative to substitution rates at all genic sites. Additionally, the absolute rates of molecular evolution identified here in *Blochmannia* are about an order of magnitude faster than those reported in *Buchnera* (Clark et al., 1999).

Relatively, fast evolution rates are expected in endosymbionts because of their life histories; insect endosymbionts' asexuality and propensity to undergo regular bottlenecks because of their mode of inheritance lead to small effective populations sizes and relatively fast evolution (Mira & Moran, 2002; Wernegreen, 2002). As such, endosymbionts also have faster evolutionary rates compared with their free‐living relatives, including increased rates of evolution at nonsynonymous coding sites (Brown & Wernegreen, 2016; Degnan et al., 2004; Moran, 1996). Because *Blochmannia* endosymbionts are asexual and likely to have small population sizes, they may undergo rapid genetic drift and experience accelerated molecular evolution (Pettersson & Berg, 2007; Rispe & Moran, 2000; Woolfit & Bromham, 2003). Additionally, even with small population sizes, obligate endosymbionts may still be under very strong within‐host selective pressures, further accelerating their molecular evolution (Perreau et al., 2021).

Endosymbiont molecular evolution rates may vary somewhat across the genome and may have host‐lineage‐specific rates of molecular evolution (Kuo & Ochman, 2009). Indeed, we found that relative rates of molecular evolution varied somewhat across *Blochmannia* genomes (Figures 3b,c). Additionally, we found that lineage‐specific rates of *Blochmannia* molecular evolution were correlated with host rates of evolution (Figure 3d). These results are similar to those found in *Camponotus* and *Blochmannia* using a small genetic dataset (two host genetic loci and four *Blochmannia* genetic loci) (Degnan et al., 2004) and suggest that molecular evolution rates—while much faster in *Blochmannia*—are correlated between carpenter ant hosts and *Blochmannia* endosymbionts at genome‐wide scales. Correlated rates of molecular evolution between hosts and endosymbionts have also been demonstrated in (1) aphids and their *Buchnera* endosymbionts (Arab & Lo, 2021) and (2) cockroaches and their *Blattabacterium* endosymbionts (Arab et al., 2020). Overall, our results corroborate previous evidence that endosymbionts have faster rates of evolution relative to both their hosts and to their free‐living bacterial relatives, even when evolutionary rates are correlated between hosts and their endosymbionts.

### 4.3 Does host demography shape endosymbiont evolution?

Because population genomic processes are influenced by effective population size, and endosymbiont effective population size is intrinsically linked with host effective population sizes (Mira & Moran, 2002; Wernegreen, 2002), we may have the expectation that host demographic patterns partially influence endosymbiont molecular evolution. Here, we investigated whether host demography influenced four factors of endosymbiont genome evolution: (1) natural selection, (2) genetic drift, (3) genome size, and (4) patterns of gene loss.

We found no relationship between host demography and both signatures of positive selection and relaxation of selection strength in *Blochmannia* genes. In contrast, we found a positive relationship between host population sizes and shifts toward intensified selection pressures in *Blochmannia* genes (Figure 7a). In endosymbionts in general, we may expect relaxed selection relative to patterns in free‐living bacteria (Wernegreen, 2002). Indeed, selection is often identified in insect endosymbionts, but generally, only in a small fraction of genes (Alleman et al., 2018; Chong et al., 2019; Williams & Wernegreen, 2012). Based on our results (Figure 7a), it appears that shifts in selection pressures may at least in part be influenced by host demographic processes.

We also tested for an effect of host demography on patterns of endosymbiont genetic drift, gene loss, and overall genome size. Here, we found no relationship between host population sizes and these characteristics of endosymbiont genome evolution (Figure 7). We initially anticipated that endosymbiont genetic drift, and associated gene loss and genome size evolution, would occur faster in endosymbionts with small host effective population sizes. While this was not the case with the entire dataset (Figure 7), the host species with the smallest estimated population sizes—*C. laevissimus* Mackay, 2019—showed the (1) highest rate of endosymbiont genetic drift as measured by genome‐wide K_a_/K_s_ ratios, (2) most endosymbiont genes with shifts toward relaxed selective pressures relative to other endosymbiont lineages, and (3) lowest *Blochmannia* gene count (Table 1).

Additionally, we found that about half of the gene loss was phylogenetically informative (Figure 4). This suggests relatively random patterns of gene loss in the phylogeny; most gene losses lacking phylogenetic signal were singleton gene losses (Figure 4). This is consistent with previous research in *Blochmannia* endosymbionts identifying lineage‐specific gene loss largely due to relaxed selection constraints and genetic drift (Williams & Wernegreen, 2015). Similarly, in cockroach *Blattabacterium* endosymbionts, Kinjo et al. (2021) found that gene loss was lineage‐specific, and that some genes showed parallel gene loss across multiple *Blattabacterium* lineages.

While we did not find a relationship between host demography and the evolution of *Blochmannia* genome sizes, we identified associations between a combination of relaxed selection strength, genome‐wide genetic drift, and *Blochmannia* genome sizes (Figure 5). Higher genome‐wide K_a_/K_s_ ratios were strongly associated with smaller *Blochmannia* genome sizes (Figure 5b). These results suggest that genetic drift, or possibly a combination of genetic drift and relaxed selection strength, is shaping genome size reduction in the *Blochmannia* genomes sampled here. This negative association between genome‐wide K_a_/K_s_ ratios and genome size is like that identified by Kuo et al. (2009) across 42 free‐living and symbiotic pairs of bacteria and suggests a general relationship between the relative rate of nonsynonymous genetic changes and genome size reduction.

Overall, we found that host demography is associated with shifts in selection strength in *Blochmannia* genomes, but not associated with several other aspects of *Blochmannia* molecular evolution. As such, we may infer that either (1) our small sample sizes (number of species) may be precluding us from identifying weak correlations between host demography and *Blochmannia* molecular evolution, or (2) host effective population sizes may not directly reflect endosymbiont effective population sizes, leading to a lack of or weak relationship between host population sizes and endosymbiont molecular evolution. If different host species or populations have varied patterns of endosymbiont transmission population bottlenecks (Mira & Moran, 2002) or endosymbiont within‐host selection (Perreau et al., 2021), we may expect a decoupling of host and endosymbiont effective population sizes.

## 5 CONCLUSIONS

We used whole‐genome sequencing of both carpenter ant hosts and their *Blochmannia* endosymbionts to investigate the influence of host demography on symbiont molecular evolution. We identified strict codiversification of *Camponotus* hosts and their *Blochmannia* endosymbionts. *Blochmannia* genes are evolving about 30× faster than host genomes, with relatively consistent evolutionary rates across the *Blochmannia* genome. We found that some, but not all, patterns of natural selection in *Blochmannia* genomes were in part shaped by host demographic history. *Blochmannia* genome size evolution was not associated with host demography but was associated with genome‐wide estimates of genetic drift and number of genes with relaxed selection pressures.

## AUTHOR CONTRIBUTIONS

**Joseph D Manthey:** Conceptualization (equal); data curation (lead); formal analysis (lead); funding acquisition (lead); investigation (lead); methodology (lead); project administration (lead); resources (equal); writing – original draft (lead); writing – review and editing (equal). **Jennifer C Giron:** Conceptualization (equal); resources (equal); writing – original draft (supporting); writing – review and editing (equal). **Jack Hruska:** Conceptualization (equal); resources (equal); writing – original draft (supporting); writing – review and editing (equal).

## CONFLICT OF INTEREST

None declared.

## Data Availability

Data associated with this manuscript may be found in the following repositories: • Raw sequencing data (NCBI BioProject PRJNA839641) • Blochmannia genome assemblies (NCBI BioProject PRJNA839441; NCBI Genome accessions CP097749‐CP097765) • Camponotus reference genome (Dryad: to be added following manuscript acceptance in accordance with Ecology and Evolution Dryad submissions) • Code for analyses (GitHub: https://github.com/jdmanthey/camponotus_genomes1).
